# Ischemic stroke can have a T1w hyperintense appearance in absence of intralesional hemorrhage

**DOI:** 10.3389/fvets.2022.932185

**Published:** 2022-09-20

**Authors:** Philippa Weston, Sebastien Behr, Laurent Garosi, Christian Maeso, Ines Carrera

**Affiliations:** ^1^Willows Veterinary Centre and Referral Service, Linnaeus Veterinary Ltd., Birmingham, United Kingdom; ^2^Vet Oracle Teleradiology, Norfolk, United Kingdom; ^3^Department of Neurology, Ars Veterinary Hospital, Barcelona, Spain

**Keywords:** cerebrovascular accident, CVA, infarction, non-hemorrhagic, dog

## Abstract

Magnetic resonance imaging (MRI) signal changes associated with ischemic stroke are typically described as T2w and FLAIR hyperintense, and T1w isointense lesions. Intralesional T1w hyperintensity is generally attributed to either a hemorrhagic stroke, or an ischemic stroke with hemorrhagic transition, and has an associated signal void on gradient echo (GE) sequences. Cases of ischemic stroke with T1w hyperintense signal in absence of associated signal void on GE sequences have been sporadically demonstrated in human stroke patients, as well as in dogs with experimentally induced ischemia of the middle cerebral artery. This multicenter retrospective descriptive study investigates the presence of T1w hyperintensity in canine stroke without associated signal void on GE sequences. High field (1.5 Tesla) MRI studies of 12 dogs with clinical presentation, MRI features, and cerebrospinal fluid results suggestive of non-hemorrhagic stroke were assessed. The time between the observed onset of clinical signs and MRI assessment was recorded. All 12 patients had an intralesional T1w hyperintense signal compared to gray and white matter, and absence of signal void on T2^*^w GE or SWI sequences. Intralesional T1w hyperintensities were either homogenously distributed throughout the entire lesion (6/12) or had a rim-like peripheral distribution (6/12). The mean time between the recorded onset of clinical signs and MRI assessment was 3 days; however, the age range of lesions with T1w hyperintense signal observed was 1–21days, suggesting that such signal intensities can be observed in acute, subacute, or chronic stages of ischemic stroke. Follow-up was recorded for 7/12 cases, all of which showed evidence of neurological improvement while in hospital, and survived to discharge. Correlation of the age and MRI appearance of lesions in this study with similar lesions observed in human and experimental studies suggests that these T1w hyperintensities are likely caused by partial tissue infarction or selective neuronal necrosis, providing an alternative differential for these T1w hyperintensities observed.

## Introduction

Stroke or cerebrovascular accidents (CVAs) are common causes of neurological dysfunction in dogs and can be broadly classified into two categories: ischemic and hemorrhagic. Hemorrhagic strokes result from rupture of a vessel supplying the brain parenchyma, whereas ischemic strokes result from occlusion of these vessels (1–3). In ischemic stroke, arterial occlusion triggers a cascade of pathophysiological processes that occur throughout the neural tissue irrigated by the affected vessel. This results in characteristic MRI features of ischemic infarction, commonly reported as sharply demarcated intra-axial lesions confined to a neovascular territory, that are primarily affecting the gray matter due to its comparative vulnerability to ischemia (1–4). These are typically homogeneously T2w and FLAIR hyperintense and T1w isointense or hypointense relative to adjacent neuroparenchyma, demonstrating absent, or weak enhancement on T1w post-contrast images, with a hypointense signal on apparent diffusion coefficient (ADC) maps. The latter denotes restrictive diffusion in the initial stages, becoming progressively ADC hyperintense from days 4–10 due to pseudonormalization followed by facilitative diffusion (2, 3, 5, 6). In true ischemic, also known as “pale” infarction, signal void is absent on T2^*^w gradient echo (GE) sequences, differentiating ischemic from hemorrhagic stroke, or ischemic stroke with hemorrhagic transition (7).

Conversely, rupture or breakdown of vascular structures seen in hemorrhagic stroke, or ischemic stroke with hemorrhagic transition, is associated with extravasation of blood into the infarcted zone, surrounding parenchyma, ventricles, subdural, or subarachnoid space leading to the development of hematoma or diffuse hemorrhagic infiltration (8). The MRI appearance of hemorrhagic stroke is largely dependent on the age of the lesion; a variable T1w and T2w intensity can be observed due to the state of oxidation of hemoglobin present and its respective magnetic properties determined by the number of unpaired outer-shell electrons of its molecular structure (9, 10). In the more chronic stages of intracranial hemorrhage, paramagnetic methemoglobin causes T1 shortening and, subsequently, a high signal on T1w images, with an associated intralesional signal void on T2^*^w gradient echo or susceptibility-weighted imaging sequences (SWI) (3, 10–12).

Although typically associated with hemorrhagic infarcts or ischemic infarcts with hemorrhagic transition, cases of presumed ischemic stroke with T1w hyperintense signal in absence of associated signal void on T2^*^w GE or susceptibility-weighted imaging (SWI) sequences have been sporadically demonstrated in human stroke patients (13–17). Although incompletely understood, these studies hypothesize that the presence of T1w hyperintense signal within non-hemorrhagic ischemic infarction is a result of incomplete tissue infarction, selective neuronal loss, or peripheral cortical laminar necrosis (6, 13, 17, 18). Histopathologically, T1w hyperintense regions within ischemic stroke lesions have been correlated with (1) focal gemistocytic astrocytosis, (2) increase in tissue protein concentration, (3) lipid deposition within macrophages, (4) tissue calcification, and (5) paramagnetic substance deposition (13, 19).

Ischemia-related T1w hyperintensities have been observed in studies of dogs with experimentally induced ischemia of the middle cerebral artery (17). In this study, an intralesional T1w hyperintensity was observed both throughout the lesion or at the lesion periphery 8 days after the onset of induced ischemia (17). However, this study lacked both assessments by gradient echo sequences and histopathology; hence, the origin of this T1w hyperintensity in the context of stroke warrants further evaluation. Cortical T1w hyperintensities are also a feature of cortical laminar necrosis, a selective polioencephalomalacia that occurs secondary to global hypoxia or hypoglycemia (20–22). This condition, however, targets the more metabolically active layers of the cerebral cortex and, hence, does not explain the T1w hyperintensity observed stroke lesions that affect structures other than the cerebral cortex. To the authors' knowledge, no studies have described non-hemorrhagic T1w hyperintense lesions specific to dogs with territorial or lacunar strokes in a clinical setting.

Considering the discrepancy in underlying pathologies caused with hemorrhagic stroke and ischemic stroke, it is important that alternative pathological processes are considered to explain the presence of T1w hyperintense signal, other than the presence of hemorrhage (5, 23). The aims of this study were 3-fold: The main aim was to investigate the presence of T1w hyperintensity in dogs with ischemic infarcts. The second aim of this study was to investigate the relationship between T1w hyperintense non-hemorrhagic infarcts and the onset of clinical signs. The third aim was to propose theories for the pathophysiology of the hyperintensity in T1w based on previous literature. It was hypothesized that first, T1w hyperintense signal would be observed without the presence of signal void in a small population of canine patients presenting with suspected CVA, and second, these lesions would be more frequently observed in the acute to subacute stages (i.e., 1–3 weeks) following the vascular event.

## Materials and methods

This is a retrospective multicenter study performed across the databases of Willows Veterinary Centre and Referral Service (Solihull, UK), Ars Veterinaria Hospital (Barcelona, Spain), and Vet Oracle Teleradiology service. Due to the non-invasive and retrospective nature of this study, ethics approval was not necessary; however, consent for the use of data was obtained for all patients. Databases were searched for dogs undergoing MRI of the brain for a suspected stroke between 2013 and 2021, using keywords “stroke,” “cerebrovascular accident,” and “CVA.”

**Figure 1:**
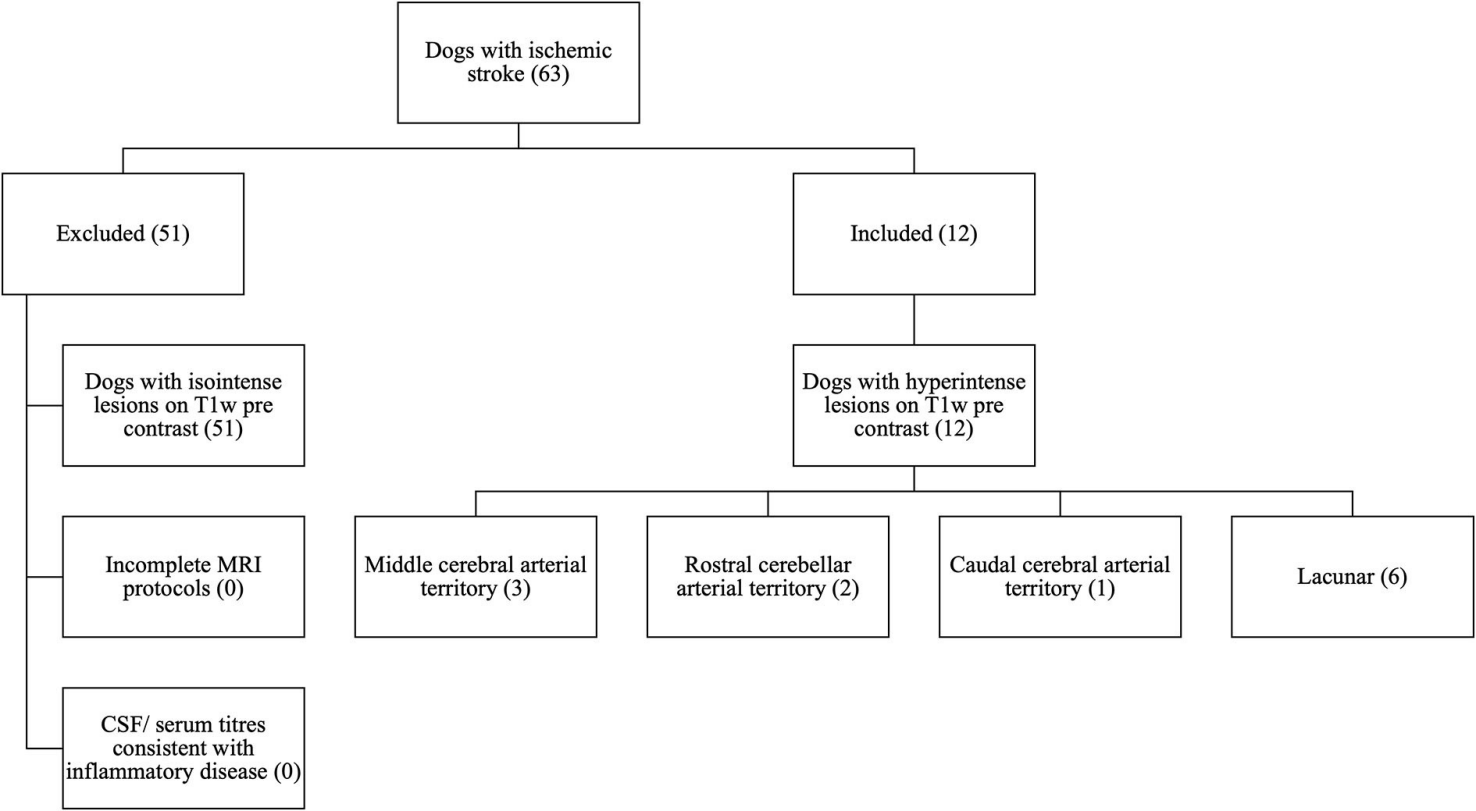
CONSORT diagram to demonstrate the selection process for patients with T1w pre-contrast hyperintense lesions from the initial 63 cases with ischemic stroke searched from clinical records.

To be included in the study, dogs must have presented for the evaluation of acute or per-acute onset of focal brain dysfunction which was non-progressive after 24 h, suggestive of stroke. Only dogs that had undergone a complete MRI study of the brain and had MRI findings compatible with brain infarct were selected (Figure 1). For the purpose of this study, a complete MRI series was defined as sagittal, dorsal, and transverse image planes and a minimum of T2-weighted (T2W), transverse pre-contrast T1-weighted images (T1W), and a T2^*^w gradient echo sequence such as T2^*^w FGE or SWI. In addition, analysis of T1w post-contrast images (IV injection of 0.1 mmol of gadolinomide/kg of body weight), fluid-attenuated inversion recovery (FLAIR), and diffusion-weighted imaging (DWI) were analyzed when available. The MRI diagnosis of presumed brain infarction was made on the basis of imaging criteria previously described in dogs (5). These included intra-axial lesions that were predominantly affecting gray matter, with occasional white matter involvement if gray matter changes are severe, and confined to a neovascular territory of a main cerebral artery (rostral cerebral, middle cerebral, caudal cerebral, rostral cerebellar, caudal cerebellar, or one of their respective branches) or a perforating artery (striate arteries, perforating arteries of the caudal communicating artery, or perforating arteries of the brainstem) (2, 3, 5).

Dogs with intracranial pathologies other than suspected stroke or with evidence of intracranial signal void on T2^*^w GE or SWI sequences were excluded from the study. Dogs with other CSF abnormalities other than those compatible with stroke were also excluded (24).

Demographic data (age, sex, breed, and bodyweight), presenting clinical signs, and physical and neurological examination findings, as performed by the attending veterinary neurologists were recorded for each case.

The onset of clinical signs was recorded from the clinical notes, and the time from the onset of clinical signs to MRI was documented where available. For the purpose of this study, lesions were dated according to the timings between the observed onset of clinical signs and the MRI assessment. Categories included early hyperacute (0 ≤ 6 h), late hyperacute (6 ≤ 24 h), acute (24 h ≤ 1 week), subacute (1 ≤ 3 weeks), and chronic (>3 weeks) (6).

Clinical findings that were documented include presence of seizures (none/isolated/cluster seizures), mentation (normal/lethargy/disorientation/depression/obtunded/stuporous), behavior (normal, altered), posture (normal/head tilt/head turn), gait (normal, ataxia, paresis/plegia) and limb(s) affected, proprioception (normal/deficits), vision (normal/unilateral or bilateral deficits), presence of cranial nerve abnormalities [normal/absent menace/absent palpebral, absent pupillary light response (PLR), absent nasal stimulation, absent gag reflex, and strabismus] and side(s) affected, hyperesthesia (yes/no), and further vestibular anomalies (none, vestibular ataxia, and nystagmus).

Cerebrospinal fluid (CSF) analysis and infectious disease analysis available were recorded. CSF findings suggestive of stroke included normal analysis results, or increased protein concentration, mild neutrophilic or mononuclear pleocytosis [30 cells/mL], xanthochromia, and hemosiderosis (5, 24). Dogs with CSF results suggestive of disease other than stroke or with evidence of infectious disease were excluded. The available point of care ELISA test (Angio Detect^TM^ IDEXX Europe B.V., Hoofed drop, The Netherlands) results were assessed, and dogs with a positive result were excluded from the analysis.

Anesthetic protocols were tailored to individual dogs by the attending anesthetist. MRI protocols and sequences varied between institutions, but all MRI examinations were completed with dogs under general anesthesia, using high-field-strength magnets: 1.5 Tesla (Hallmarq PetVet; Siemens Magnetom Sola; Canon Vantage Elan).

The MRI studies were reviewed by a European College of Veterinary Diagnostic Imaging board-certified veterinary radiologist (I.C.), and a third-year veterinary radiology resident (P.W.), on DICOM viewing software (OsiriX, Pixmeo, Switzerland). Reviewers were aware of the history, patient signalment, clinical, and neurological examination findings. Images were reviewed individually followed by consensus evaluation.

On the basis of the MRI findings, infarcts were classified for each dog as (1) location within the brain (telencephalon, thalamic/midbrain, pons/medulla, cerebellum, and multifocal) and (2) infarct type (territorial, lacunar, or watershed). Territorial infarcts were defined as infarcts occupying the vascular territory of one of the main arteries of the brain. Lacunar infarcts were defined as subcortical infarcts limited to the vascular territory of an intraparenchymal superficial or deep perforating artery, and watershed infarcts were defined as an infarct in the boundary zone between large artery territories (5). (3) Vascular region was affected; for territorial lesions, this included rostral cerebral, middle cerebral, caudal cerebral, rostral cerebellar, caudal cerebellar, or one of their primary branches (3). For lacunar lesions, this included the striate arteries, proximal and distal perforating artery arising from the caudal communicating artery, caudal perforating arteries originating from the basilar bifurcation, and paramedian branches arising from the proximal portion of the caudal cerebral artery (25–27). (4) Imaging characteristics assessed included (a) signal intensity on T1w, T2w, FLAIR, SWI or T2^*^w GE, sequences, (b) appearance of lesion on T1w and T2w (heterogenous/homogeneous), (c) lesion margination (poorly defined/moderate/well-defined), (d) presence of contrast enhancement (none/mild/moderate/severe), (e) pattern of contrast enhancement (uniform/non-uniform/focal/rim-like), (f) presence of perilesional edema [none/perilesional (<10 mm)/extensive (>10 mm)], (g) associated mass effect (none/subarachnoid CSF signal loss/midline shift/ventricular distortion/brain herniation) (subfalcine, transtentorial, foramen magnum), hydrocephalus, or (h) presence of parenchymal atrophy (none/mild/moderate/severe). Signal intensity on diffusion-weighted imaging (DWI) and ADC values was recorded where available. Imaging characteristics as described are summarized in Table 1, and an example of MRI sequence parameters used is provided in Table 2.

**Table 1:** Summary of imaging features recorded.

**MRI feature**	**Description**	
Location	Telencephalon/thalamic/midbrain/pons/medulla/cerebellum/multifocal	
Infarct type	Territorial	Infarcts occupying the vascular territory of one of the main arteries of the brain (5).
	Lacunar	Subcortical infarcts limited to the vascular territory of an intraparenchymal superficial or deep perforating artery (5).
	Watershed	An infarct in the boundary zone between large artery territories (5).
Vascular region affected	Territorial	Rostral cerebral, middle cerebral, caudal cerebral, rostral cerebellar, caudal cerebellar or one of their primary branches (3).
	Lacunar	Striate arteries, proximal and distal perforating artery arising from the caudal communicating artery, caudal perforating arteries originating from the basilar bifurcation, and paramedian branches arising from the proximal portion of the caudal cerebral artery (25–27).
Imaging characteristics	Signal intensity	Intensity on T1w, T2w, FLAIR, SWI or T2*w GE, sequences
	Appearance of signal	Heterogeneous/homogeneous
	Lesion margination	Poorly defined/moderate/well-defined
	Contrast enhancement	None/mild/moderate/severe
	Pattern of contrast enhancement	Uniform/non-uniform/focal/rim-like
	Perilesional oedema	None/perilesional (<10 mm)/extensive (>10 mm)
	Mass effect	None/subarachnoid CSF signal loss/midline shift/ventricular distortion/brain herniation (sub-falcine, trans-tentorial, foramen magnum), hydrocephalus
	Parenchymal atrophy	None/mild/moderate/severe
	DWI/ADC intensity	Hypointense/Hyperintense

**Table 2:** MRI acquisition parameters used.

**Parameter**	**T1w (pre + post contrast)**	**T2w**	**FLAIR**	T2*w GE	**SWI**
TE (ms)	15	105	120	9	30
TR (ms)	600	5,000	8,000	500	3,500
Number of Signals Averaged	2	2	2	2	1
Echo train length	3	13	19	1	1
Flip angle (°)	90	90	90	20	10
Slice thickness (mm)	3.0	3.0	2.8	3.0	1
Interslice gap (mm)	3.3	3.3	3.3	3.3	0.5
Acquisition matrix	224\192	240\224	192\160	224\216	288\240
Field of View (mm^2^)	100	100	100	100	100

Data regarding treatment protocols were recorded for each patient. Survival was defined as the time from imaging diagnosis (MRI scan). Follow-up assessment was obtained from medical records where available.

## Results

Sixty-three dogs met the inclusion criteria, 12 of which had lesions with T1w hyperintense signal without associated signal void on T2^*^w GE or SWI sequences (Figure 1). The median age was 7.5 years (range 5 months−11.5 years). Breeds included Greyhound (2), French Bulldog (2), Labrador Retriever (2), Cocker Spaniel (1), English Springer spaniel (1), Lurcher (1), West Highland White Terrier (1), Spanish Hound (1), and a Border Collie (1). Patients were female neutered (6), male neutered (3), female entire (2), and male entire (1).

Presenting clinical signs included acute or per-acute onset vestibular abnormalities (7/12), generalized seizures (4/12), collapse (2), unilateral blindness (2), and gait abnormalities (1). As detailed in Table 3, abnormalities documented on neurological examination by the attending neurologist included head tilt (7), proprioceptive deficits (6), head turn (3), circling (3), ataxia (3), tetraparesis (2 non-ambulatory, 1 ambulatory), hemiparesis (1), unilateral blindness (2), rotatory nystagmus (1), and vertical nystagmus (1). Documented cranial nerve abnormalities included absent or reduced menace (3 unilateral and 1 bilateral), absent pupillary light reflex (PLR) (1 unilateral), and absent vestibulocochlear (2) reflex, alongside left ventral strabismus, ptosis, and enophthalmos in one case. Hyperesthesia was not documented in any patient.

**Table 3:** Clinical presentation and neurological exam findings.

	**Seizures (none/isolated/ cluster)**	**Mentation (normal/ lethargic/ disorientated/ depressed/ obtunded/ stuporous)**	**Behavior (normal/ altered)**	**Circling**	**Posture (normal/head tilt/head turn)**	**Gait (normal/ataxia/ paresis/plegia)**	**Proprioception (normal/deficits)**	**Vision (normal/ unilateral/bilateral deficits)**	**Cranial nerve deficits (normal/absent menace/absent palpebral, absent PLR, absent nasal stimulation, absent gag reflex)**	**Hyperaesthesia (yes/no)**	**Other vestibular anomalies (none, vestibular ataxia, nystagmus)**	**Lesionlocation**	**Presenting complaint**
1	None	Normal	Normal	Normal	Left sided head turn, left sided head tilt	Cerebellar ataxia	Normal	Normal	Absent vestibulocochlear reflex	No	Absent vestibulocochlear reflex	Left rostral cerebellum	Vestibular abnormalities
2	None	Normal	Normal	Normal	Normal	Left hemiparesis	Absent left sided paw placement and hopping	Left unilateral blindness	Left PLR and menace absent	No	None	Right caudate nucleus	Left unilateral blindness and gait changes
3	None	Disorientated	Normal	Normal	Left sided head tilt, left sided head turn	Ataxia	Increased tone right thoracic and pelvic limbs.	Normal	Reduced left sided vestibulo-occular reflex. Left sided mydriasis with positive PLR.	No	Rotatory nystagmus and vestibular ataxia	Rostro medial aspect of the right tegmentum	Vestibular signs
4	Isolated, generalized.	Normal	Normal	Normal	Right sided head tilt, right sided head turn	Falling to the right. Ataxia.	Absent right paw placement	Normal	Normal	No	Vestibular ataxia	Right rostral cerebellum - entire right hemisphere and right half of cerebellar vermis	Seizures, vestibular abnormalities
5	None	Normal	Normal	Normal	Right sided head tilt.	Non-ambulatory tetra paresis	Normal	Normal	Normal	No	Vestibular	Caudomedial aspect of the left thalamic lobe, and rostral aspect of the left tegmentum	Collapse
6	None	Normal	Normal	Normal	Right sided head tilt.	Non-ambulatory tetra paresis	Absent right paw placement	Normal	Normal	No	Vestibular	Left temporal and parietal lobe	Collapse
7	Cluster, generalized	Disorientated	Normal	Circling to the left	Left sided head tilt	Normal	Delayed right sided paw placement	Normal	Absent right sided menace.	No	Vestibular	Left cerebral hemisphere	Seizures
8	Cluster, generalized	Depressed	Normal	Normal	Normal	Normal	Normal	Normal	Bilaterally reduced menace	No	None	Right temporal, occipital and piriform lobe	Seizures
9	None	Normal	Normal	Normal	Left sided head tilt and falling to the left	Falling to the left	Normal	Normal	None	No	Vertical nystagmus	Caudomedial aspect of right thalamic lobe	Vestibular
10	2 isolated, generalized	Normal	Normal	Normal	Normal	Normal	Normal	Normal	Normal	Normal	Normal	Right occipital cortex	Seizures
11	None	Depressed	Normal	Circling to the left	Normal	Ambulatory tetraparesis	Increased tone all four limbs	Normal	Normal	Normal	Normal	Left caudate nucleus	Gait abnormalities
12	None	Normal	Normal	Circling to the left	Normal	Normal	Normal	Right unilateral blindness	Absent right menace, left ventral strabismus. Left enophthalmos/ptosis	No	None	Left thalamus	Circling

The exact time between the onset of clinical signs observed and the MRI assessment was recorded in 7/12 patients. All remaining (5/12) patients were scanned within 1 week of the onset of clinical signs. The mean time between the MRI scan and observed onset of clinical signs in this patient cohort was 3 days, with a range of 24 h to 21 days. As defined above, at the time of MRI scan, 9/12 patients had acute stroke, 2/12 had chronic stroke, and 1/12 had a subacute lesion. An example of the MRI sequence parameters used is provided in Table 2.

**Figure 2:**
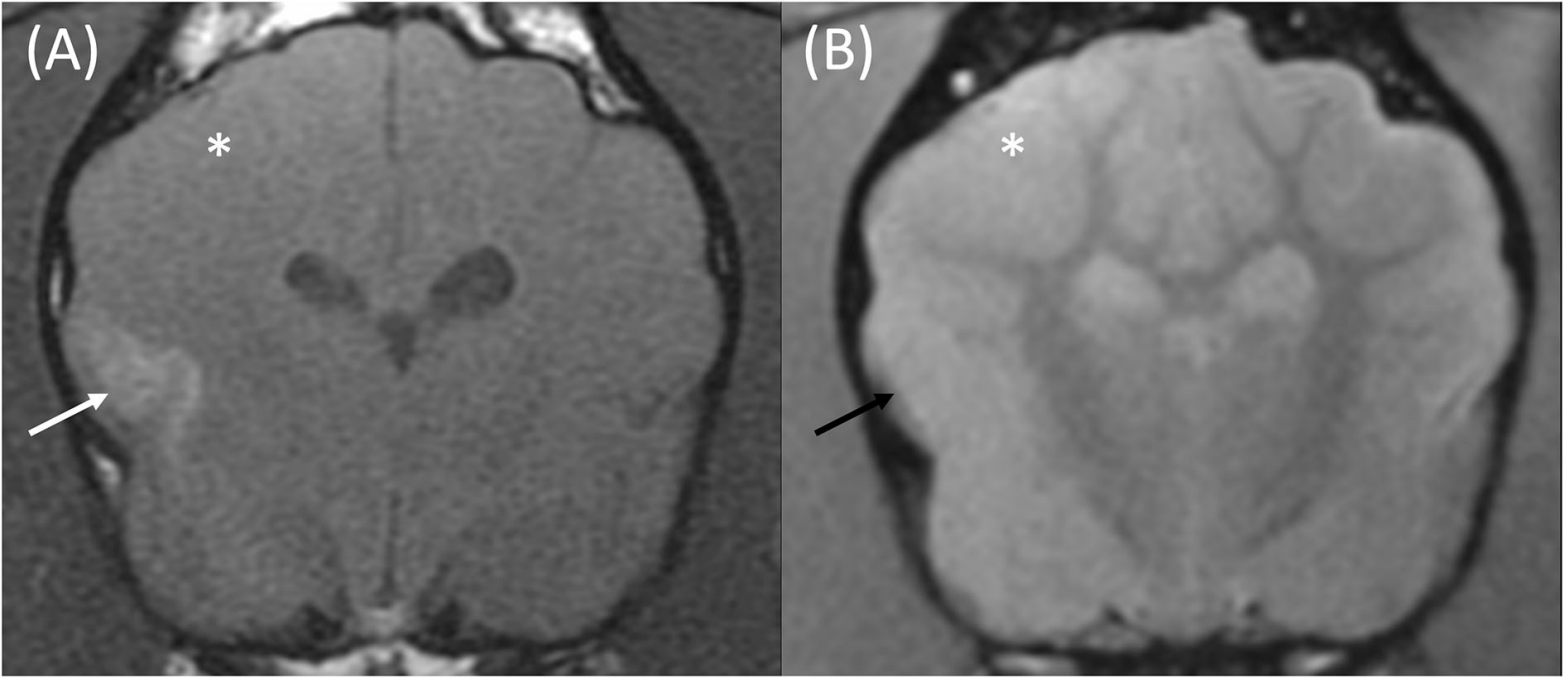
Transverse T1w pre-contrast **(A)** and T2*w GE **(B)** images at the level of the caudate nuclei in an 8-month male entire French Bulldog that presented with acute onset generalized seizures. The MRI was performed 24 h following the observed seizures. These findings are compatible with an ischemic stroke of the right middle cerebral artery. The T1w pre-contrast image **(A)** demonstrates a poorly defined T1w hyperintense signal affecting the cortical gray matter of the suprasylvian gyrus and pseudosylvian fissure (white arrow). No signal void is seen on the corresponding T2*w GE sequence (**B**, black arrow). The gray matter of the right parietal and temporal lobes is swollen, and the gyri are effaced on both sequences (white asterisk).

**Figure 3:**
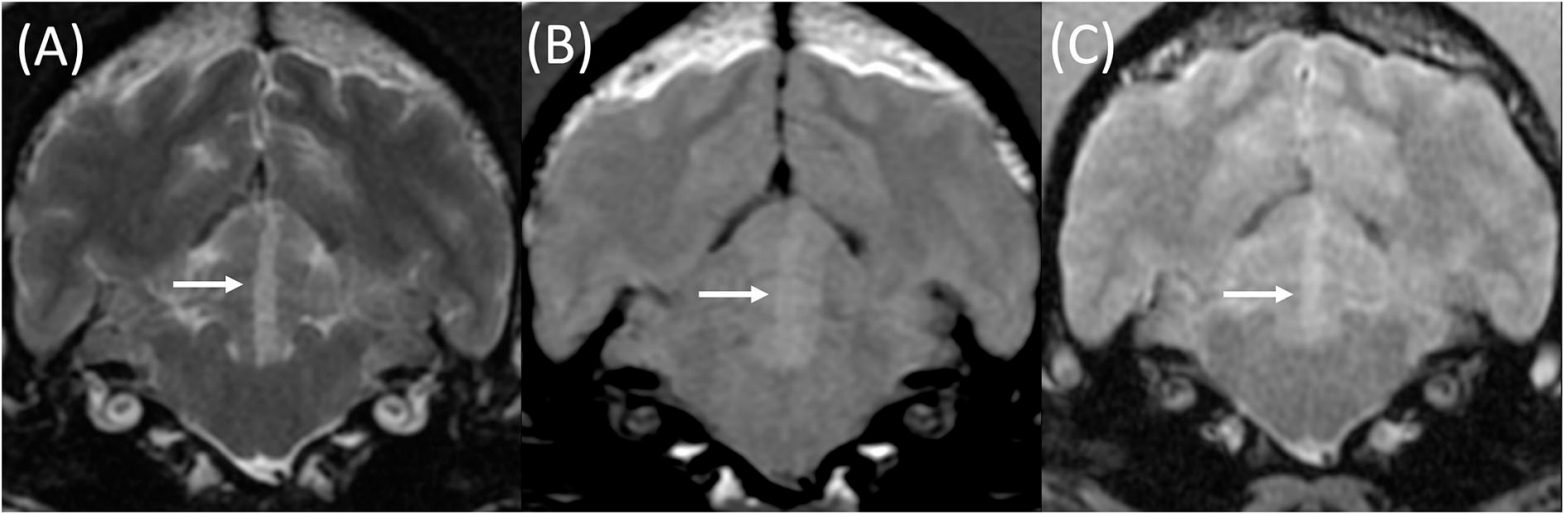
Transverse T2w **(A)**, T1w pre-contrast **(B)**, and T2*w GE **(C)** images at the level of the rostral cerebellum in an 8-year-old female neutered Cocker Spaniel with acute onset cerebellar ataxia. The MRI was performed 3 days following the onset of clinical signs. A well-demarcated lesion is present within the left rostral cerebellum (white arrows). This is compatible with a territorial infarction of the median branch of the left rostral cerebellar artery. This lesion is homogeneously T2w **(A)** and T1w pre-contrast **(B)** hyperintense to gray and white matter, without signal void on T2*w GE sequences **(C)**.

**Figure 4:**
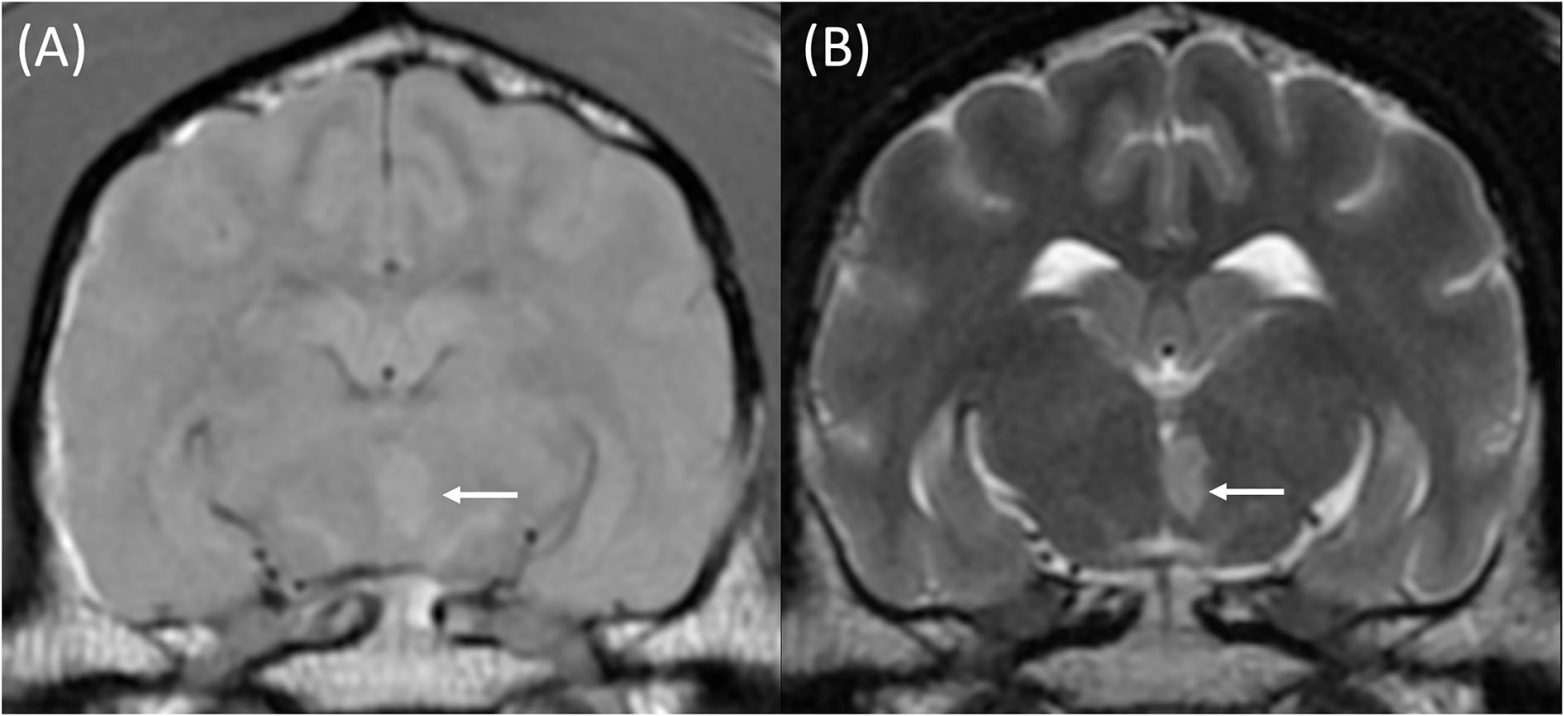
T1w pre-contrast **(A)** and T2w **(B)** transverse images at the level of the piriform lobes of an 11-year-old female neutered Labrador Retriever that presented with acute onset left-sided central vestibular deficits. The MRI was performed within 3 days following the onset of clinical signs. A well-defined T1w pre-contrast and T2w hyperintense lesion is centered on the caudomedial aspect of the left thalamic lobe (A + B, white arrows). This was most likely compatible with a territorial infarction of the left caudal perforating artery.

Upon MRI examination, all patients had a solitary lesion confined to the anatomical territory of a cerebral or cerebellar artery, and all lesions predominantly affected gray matter (12/12). These included 6/12 territorial lesions centered on territories of the middle cerebral (3), rostral cerebellar (2), and caudal cerebral arteries (1), alongside 6/12 lacunar infarcts centered on the irrigated region if the caudal perforating (3), lateral striate (2), and proximal perforating (1) arteries. Examples of such lesions are demonstrated in Figures 2–4, and a summary of imaging findings is detailed in Table 4.

**Table 4:** Findings of MRI assessment.

	**Lesionlocation**	**Infarct type**	**Territoryaffected**	**Gray/white matter affected**	**Definition**	**T1w hyperintensity - degree**	**T1w hyperintensity - distribution**	**T2w Intensity**	**FLAIR intensity**	Signal void on T2*w/SWI	**ADC/DWI**	**Contrastenhancement**	**Perilesionaloedema**	**Mass effect**	**Parenchymal atrophy**	**Other**	**Time from clinical onset to MRI (days)**	**Age category**
1	Left rostral cerebellum	Territorial	Median branch of left rostral cerebellar artery	Gray matter	Well-defined	Moderate	Diffuse + homogeneous throughout	Hyperintense (homogeneous)	Hyperintense	Absent	Not performed	Non-enhancing	Absent	Absent	Absent	None	3	Acute
2	Right caudate nucleus	Lacunar	Right lateral striate artery (from middle cerebral artery)	Gray matter	Poorly defined	Mild	Diffuse + heterogeneous throughout lesion	Hyperintense (homogeneous)	Hyperintense	Absent	Not performed	Mild, heterogeneous	Moderate- along corona radiata	Slight left sided midline shift of falx cerebri and mesencephalic aqueduct	Absent	None	6	Acute
3	Rostromedial aspect of the right tegmentum	Lacunar	Right caudal perforating artery	Gray matter	Well-defined	Moderate (relative to gray and white matter)	Moderate peripheral T1 hyperintense, mild central T1w hyperintense	Hyperintense (homogeneous)	Hyperintense	Absent	Not performed	Mild peripheral contrast enhancement	Mild	Absent	Absent	CT of thorax and abdomen - small cholelith	1	Acute
4	Right rostral cerebellum - entire right hemisphere and right half of cerebellar vermis	Territorial	Right rostral cerebellar artery (intermediate and medial branches)	Gray matter	Well-defined	Mild	Diffuse + homogeneous throughout	Hyperintense (homogeneous)	Hyperintense	Absent	DWI hyperintense. ADC hypointense (restrictive diffusion)	Mild heterogeneous enhancement throughout the lesion	Mild	Absent	Absent	None	1	Acute
5	Caudomedial aspect of the left thalamic lobe, and rostral aspect of the left tegmentum	Lacunar	Left caudal perforating artery	Gray matter	Well-defined	Moderate	Diffuse + homogeneous throughout	Hyperintense (homogeneous)	Hyperintense	Absent	DWI hyperintense. ADC hypointense (restrictive diffusion)	Non-enhancing	Absent	Absent	Absent	None	<1 week	Acute
6	Left temporal and parietal lobe	Territorial	Left middle cerebral artery	Gray matter	Well-defined	Moderate	Peripheral- left sylvanian gyrus, marginal sulcus and pseudosylvanian fissure of the left temporal and parietal lobes	Hyperintense (homogeneous)	Hyperintense	Absent	Not performed	Mild enhancement at rostral periphery	Absent	Absent	Absent	None	1	Acute
7	Left cerebral hemisphere	Territorial	Left middle cerebral artery	Gray matter	Well-defined	Moderate	Peripheral	Hyperintense (homogeneous)	Hyperintense	Absent	DWI hyperintense. ADC isointense (Pseudonormalisation)	Moderate peripheral cortical enhancement	Absent	Absent	Absent	Small T1w isointense chronic lacunar infarct left rostral tectum and caudal left thalamus.	<1 week	Acute
8	Right temporal, occipital and piriform lobe	Territorial	Right middle cerebral artery	Gray and white matter	Poorly defined	Severe	Severe along the peripheral inner most cortex adjacent to the right psuedosylavanian fissure. Moderate and slightly heterogeneous throughout the adjacent gray matter.	Hyperintense (homogeneous)	Hyperintense	Absent	DWI hyperintense. ADC hypointense (Restrictive diffusion).	Non-enhancing	Moderate	Mild left sided displacement of mesencephalic aqueduct and falx cerebri	Absent	None	<1 week	Acute
9	Caudomedial aspect of right thalamic lobe	Lacunar	Right caudal perforating artery	Gray matter	Well-defined	Mild	Diffuse + homogeneous throughout	Hyperintense (homogeneous)	Hyperintense	Absent	Not performed	Mild homogeneous enhancement	Absent	Absent	Absent	None	<1 week	Acute
10	Right occipital cortex	Territorial	Right caudal cerebral artery	Gray matter	Well-defined	Mild	Diffuse + homogeneous throughout	Hyperintense (homogeneous)	Hyperintense	Absent	DWI isointense. ADC isointense (Pseudonormalisation).	Non-enhancing	Absent	Absent	Absent	None	8	Subacute
11	Left caudate nucleus	Lacunar	Left lateral striate artery	Gray matter	Well-defined	Moderate	Peripheral	Hyperintense (homogeneous)	Hyperintense	Absent	DWI isointense. ADC isointense (Pseudo-normalization).	Mild homogeneous enhancement	Mild	Mild - ventral compression of the left lateral ventricle	Absent	Further chronic infarcts within the right caduate nucleus and thalamus - without enhancemnet of T1w hyperintensity.	20	Chronic
12	Left rotromedial thalamus	Lacunar	Left proximal perforating artery from the caudal communicating artery	Gray matter	Well-defined	Severe	Peripheral	Hyperintense (homogeneous)	Hyperintense	Absent	DWI hyperintense. ADC hyperintense (Facilitative diffusion).	Mild and homogeneous throughout the lesion	Moderate	Mild lateral ventricular oedema	Absent	T2w hyperintense striation on the left medulla	21	Chronic

All lesions (12/12) were homogeneously T2w and FLAIR hyperintense relative to both gray and white matter, and none had appreciable signal void on T2^*^w or SWI sequences. All lesions (12/12) were T1w hyperintense relative to both gray and white matter. When T1w signal hyperintensity was graded in comparison with the basal ganglia, 4/12 were described as mild, 6/12 moderate, and 2/12 severely T1w hyperintense. In terms of chronicity, the lesions with mild T1w hyperintensity were both acute (3) and subacute (1). Of the six (6/12) with a moderate T1w signal, 5/12 of these patients had acute lesions, and 1/12 had a chronic lesion. The severe T1w hyperintense signal noted in 2/12 was both acute (1) and chronic (1) in nature. In a large proportion of cases (6/12), the T1w hyperintense signal was distributed throughout the lesion, with a homogeneous (5/12) or heterogeneous pattern (1/12). Interestingly, in 6/12 cases, the observed T1w hyperintense signal had an almost linear, peripheral distribution, with a T1w hyperintense-isointense center. This was circumferential in three cases of lacunar infarction, located in the rostral thalamus, tegmentum, and caudate nucleus. In three cases of stroke of the middle cerebral artery, peripheral T1w hyperintensities were noted along the overlying cerebral cortices. In two of these cases, this most severely affected the outermost aspect of the cerebral cortex, in particular along the sylvian gyrus, marginal sulcus, and pseudosylvian fissure of the temporal and parietal lobes. In the third case, the peripheral T1w hyperintensity was most severe along the innermost cortex adjacent to the right pseudosylvian fissure, with a mild and heterogeneous T1w hyperintense signal throughout the remainder of the lesion.

DWI sequences and apparent diffusion coefficient (ADC) maps were acquired in 7/12 cases. In 3/7 cases, lesions were hyperintense on DWI and hypointense on ADC, suggestive of restrictive diffusion. All of these lesions were acute in nature, corresponding with predominant cytotoxic edema seen in acute stages of infarction. Three (3/7) cases had lesions that were DWI hyperintense and ADC isointense relative to the contralateral gray matter, and these lesions were defined as acute (<1 week), subacute (8 days), and chronic (20 days) which may suggest an early stage of pseudonormalization, which is typically observed from 4 to 10 days of the ischemic event, secondary to cell lysis and subsequent facilitative diffusion (2). In one case, the infarct was ADC and DWI hyperintense, suggestive facilitative diffusion among cells due to a predominance of vasogenic edema. This corresponded with the chronicity of the lesion age (21 days).

All cases were assessed on T1w post-contrast, and 8/12 had a mild contrast enhancement. In 2/12, this was mild and heterogeneously distributed throughout, in 3/12, this was mild and homogeneously distributed throughout, and in 3/12, mild peripheral contrast enhancement was noted. The cases where peripheral contrast enhancement patterns were observed correlated with the observed distribution of the hyperintense signal on T1w pre-contrast images. Contrast enhancement was not evident in 4/12 cases.

The majority of lesions were well-demarcated (10/12). One was poorly defined due to extensive perilesional edema which extended into the surrounding white matter of the corona radiata and internal capsule. The second poorly defined lesion was centered on the caudate nucleus and, again, had a moderate amount of perilesional edema noted on FLAIR sequences. Mild perilesional edema was affecting four further lesions. The majority of cases had no associated mass effect (9/12). Three (3/12) cases exhibited mild mass attributable to the presence of perilesional edema, resulting in midline shift of the falx cerebri and mesencephalic aqueduct (2), and mild ventral compression of the left lateral ventricle (1). Parenchymal atrophy was not observed in any case.

Two cases had concomitant, likely chronic infarcts, appearing as small, discrete foci that were T2w hyperintense and T1w isointense to gray matter, with no contrast enhancement, perilesional edema, or signal void on T2^*^w GE or SWI sequences. One was in the territory of the left caudal perforating artery (left caudal thalamus) and the other in the right caudate nucleus. Neither of these concurrent lesions documented clinical signs attributable to their location, hence were presumed to be incidental findings at the time of image evaluation and at the time of image review.

Supportive treatment was provided to the majority of cases (7/12) which included aspirin (3) and clopidogrel (2), omeprazole (1), and anti-epileptic drugs, (1) while 5/12 had no treatment documented. The latter five cases showed initial neurological improvement while hospitalized, however, were subsequently lost to follow-up. The remaining 7/12 survived to discharge with clinical and neurological improvement documented in the medical records. Of these, further follow-up obtained in three cases showed resolution of neurological signs at 7 days (1), 1 month (1), and 1 year (1).

## Discussion

This study documents the presence of T1w hyperintense signal in ischemic stroke without evidence of hemorrhage on T2^*^w GE or SWI sequences. This characteristic has been documented in human stroke patients (13–15, 28); however, to the authors' knowledge, this is the first report of this kind in clinical veterinary literature.

Twelve patients in this study had an intralesional T1w hyperintense signal compared to gray and white matter, and absence of signal void on T2^*^w GE or SWI sequences. When the distribution of the T1w hyperintense signal throughout the ischemic lesions was assessed, two clear patterns were observed; signal was either distributed throughout the entire lesion (6/12), or a more linear T1w hyperintensity was observed at the periphery of the lesion (6/12). This variation was observed in both lacunar (3/6) and territorial (3/6) lesions. The distribution of T1w hyperintense signal may reflect the inherent tissue susceptibility to hypoxia, or perfusion dynamics observed in ischemic stroke lesions (28–30). Consideration of the pathophysiology observed in ischemic stroke may help to explain the evolution of such signal change. Sequel to arterial occlusion, three separate regions of hypoperfused tissue are observed throughout the irrigated region, namely, the oligemic region, penumbra, and the infarct core (31). Oligemia refers to asymptomatic hypoperfused tissue that recovers without the need for reperfusion treatment and correlates with cerebral blood flow values that are reduced, but greater than the ischemic threshold of tissue (11). Ischemia refers to symptomatic hypoperfused tissue where cerebral blood flow is reduced below the cell's ischemic threshold (31). Within this, there is the infarct core of neural tissue that dies rapidly due to severe hypoperfusion, surrounded by the penumbra of salvageable hypoperfused tissue that has retained blood flow and metabolic function from collateral vasculature (31).

Less commonly reported sequelae of ischemic infarction include partial or incomplete infarction, selective neuronal necrosis (SNN), and cortical laminar necrosis (CLN) (17, 22, 28, 29, 32). These processes have demonstrated T1w hyperintense signal without corresponding hemorrhage in human stroke patients and experimental animal models, serving as potentially viable differentials for the hyperintense T1 signal observed in this patient cohort. All processes pertain to selective necrosis of neurons with preservation of less metabolically active cells, namely, endothelial cells, microglia, astroglia, and neurons, the death of which is conversely typical of tissue pannecrosis (13–16, 28). The terms, partial infarction and SNN, are frequently used interchangeably (28–30). Partial infarction is reported as a transient process in the acute stages of infarction and is seen in both transient ischemic attack or sequel to the development of pannecrosis (28, 33). Conversely, SNN is thought to persist into the chronic stages of infarction, potentially contributing to post-stroke cognitive decline observed in human neurodegenerative disorders such as vascular dementia (28, 32, 34). Both SNN and partial infarction have demonstrated similar patterns of delayed T1w hyperintensity within the affected tissue when studied in human patients and experimental animal models (13, 15, 17). Partial infarction and SNN have been identified histopathologically throughout the border zone of tissue infarction and throughout the penumbra on perfusion studies, which relates to the reduced cerebral perfusion throughout this region (35–39). However, SNN and partial infarction have also been observed throughout the entire lesion in some histopathological studies, resulting in a T1w hyperintense signal that is observed throughout the lesion entirety (28). Half (50%) of the patients in this study demonstrated a T1w hyperintensity throughout the entire lesion, whereas the remaining 50% had a peripherally distributed T1w hyperintense signal observed, meaning that when considering the conventional imaging features alone, both processes could account for the varied signal distribution observed.

The age of the lesions in our study also reflects temporal changes reported in studies of partial infarction and SNN. Histopathological studies in rats have demonstrated partial infarction to occur within 12 h following brief (10–25 min) occlusion of the middle cerebral artery (30), whereas experimental canine stroke models have observed T1w hypointense-isointense signal attributable to selective neuronal necrosis throughout the lesion within the first 3 days, a peripheral T1w hyperintense signal at 8 days, returning to a homogeneous T1w hypointense-isointense signal at 35 days (17). Similarly, T1w hyperintensities have been observed between 7 and 14 days in both humans and rats with neuronal necrosis secondary to acute cerebral infarction (14, 15). All patients in this cohort underwent MRI during the acute, subacute, or chronic stages of the ischemic event, with the mean age of the lesion being 3 days, and the range of lesion age being 1–21 days. Timings also correlated with the DWI/ADC signal intensity observed, where the majority of patients demonstrated DWI hyperintense and ADC hypointense signal, suggestive of restrictive diffusion typically seen within the first 4–10 days of an ischemic event (2, 5, 40). Given variation in timings reported for partial infarction, from 12 h, and selective neuronal necrosis, from 8 to 35 days, it is plausible that either process may account for the observed changes in our patient cohort.

Ostensibly, the presence of CLN could explain the peripheral T1w hyperintense signal observed in 3/6 cortical lesions of this study. Cortical laminar necrosis is a more specific subtype of infarction seen after global or focal ischemia that affects the cerebral cortex. It is a selective polioencephalomalacia occurring in response to transient or partial hypoxia or hypoglycemia, leading to necrosis of more metabolically active regions throughout cortical layers 3 and 5 (20, 34, 41, 42). T1-weighted (T1W) curvilinear hyperintense lesions affecting the gyral anatomy of the cerebral cortex are pathognomonic characteristics of cortical laminar necrosis on MRI imaging of human patients (22, 32). However, cortical laminar necrosis typically appears in the acute and subacute stages, frequently occurring at 8 days after the suspected hypoxic event, and being most pronounced at 1 month (32). These changes are somewhat more chronic than the majority of cortical lesions in our study, where indeed, MRI was performed within 24 h of the ischemic episode in two of the three cases of cerebral infarction in this patient cohort. Furthermore, cortical laminar necrosis is frequently limited to the gyral crests in cases of global ischemia, whereas in the three cases observed here, the linear T1w hyperintensity was observed along both sulcal and gyral margins overlying focal territorial regions of affected tissue (42). However, as cases of CLN have been observed in patients with regional ischemia, CLN cannot be completely excluded as a differential for cases affecting the cerebral cortex without histopathological analysis.

The exact pathophysiological mechanism for these overarching concepts of partial infarction and SNN is yet to be established (28). Histopathologically, these areas of selective neuronal death have demonstrated increased fat deposition, tissue calcification or mineralization, and increased protein accumulation resulting from glial responses and ultrastructural cellular alterations, all of which can attribute to T1 shortening (6, 18). Accumulation of minerals such as copper, manganese, and calcium within ischemic tissue can result from increased cellular oxidative stress and activation of various metabolic enzymes. These enzymes deposit paramagnetic mineral material throughout affected tissue, potentiating T1 shortening (14, 16). However, in line with our findings, the absence of mineral attenuation on CT studies of human patients and absence of associated signal void on SWI or T2^*^w MRI may oppose this theory of intralesional mineralization (13). Furthermore, histopathological assessment of human brains with T1w hyperintense ischemic lesions has failed to identify tissue mineralization, inferring that more than one pathophysiological mechanism may be responsible (15). Gemistocytic astrocytosis has been observed in T1w hyperintense lesions associated with SNN (16). Gemistocytic astrocytes, which usually appear in response to acute brain injury, have protein hydration layer which also results in T1 shortening (13). Post-ischemic ultrastructural changes in astrocyte cytoplasm, vacuolization, and proliferation of cellular organelles also increase the cellular protein content which can contribute to the hyperintense T1w tissue signal (16). Phagocytosis of damaged tissue by macrophages (microglia) and resultant intracellular lipid accumulation within microglia may also be responsible for the observed T1w hyperintensity due to increased fat content of the affected tissue (15).

Further histopathological studies are warranted to confirm the presence of partial infarction or selective neuronal necrosis within the T1w hyperintense lesions observed. Furthermore, longitudinal prospective studies in clinical patients documenting temporal changes on MRI would be helpful to establish the evolution of these lesions and to provide further understanding of their prognostic value. Similarly, studies correlating these non-hemorrhagic T1w hyperintensities with perfusion-weighted imaging to investigate the flow dynamics within the affected region are warranted to further out understanding of the pathophysiological mechanisms involved.

This study is limited first by the small patient sample size. However, this condition is scarcely reported in human patients and is rarely seen in veterinary patients, meaning that cases with these changes are challenging to recruit (25, 26). Furthermore, follow-up MRI assessment is rarely performed in dogs, in particular if a positive response is observed clinically, as it is not ethically justifiable for such cases a second general anesthetic. This means that the temporal evolution of stroke in clinical studies is difficult to evaluate, and if such T1w hyperintensities are transient or delayed features, may not be present at the time of the MRI assessment. Nevertheless, future studies across a larger patient cohort are warranted to establish the temporal evolution of T1w hyperintense signal seen in non-ischemic stroke in canine patients. This study is also limited by its retrospective nature and multicenter design, meaning that study protocols cannot be unified, and in particular the strength of the MRI magnet and gradient coils is not uniform between centers. Furthermore, the clinical relevance of these lesions is difficult to ascertain as many patients in this cohort were lost to follow-up. Lastly, histopathological assessment was not available for any patient in this study, which is largely reflective of the inclusion criteria and nature of CVA, whereby none of the patients in our study were euthanized throughout the time of patient recruitment. Future studies across a larger patient cohort assessing perfusion parameters, clinical outcomes, and follow-up MRI assessment are warranted for further investigation.

## Conclusion

Non-hemorrhagic T1w hyperintense signal observed in ischemic stroke has, to date, been rarely reported in canine patients. These lesions may be seen both within cortical territorial infarcts, or smaller lacunar infarcts throughout the non-cortical parenchyma, and can have either a homogeneous or peripheral distribution. Although the precise pathophysiology of this signal remains unknown, the presence of a T1w hyperintense signal in absence of signal void may raise suspicion of partial tissue infarction or selective neuronal necrosis, rather than early hemorrhagic transition in stroke cases.

## Data availability statement

The datasets presented in this study can be found in online repositories. The names of the repository/repositories and accession number(s) can be found in the article/supplementary material.

## Ethics statement

Ethical review and approval was not required for the animal study because this study is retrospective in nature, hence all procedures performed were for diagnostic or therapeutic purposes deemed necessary by the handling clinician at the time of presentation. Furthermore, consent was obtained from each owner for the use of the patients images and medical records in research studies upon admission of the patient. Written informed consent was obtained from the owners for the participation of their animals in this study.

## Author contributions

IC conceived of the presented idea. PW and IC performed the image evaluation. PW wrote the manuscript. All authors contributed to the article and approved the submitted version.

## Funding

Linnaeus Veterinary Limited supported the costs of the Open Access Publication Charges.

## Conflict of interest

Authors PW, SB, and IC were employed by Linnaeus Veterinary Ltd. The remaining authors declare that the research was conducted in the absence of any commercial or financial relationships that could be construed as a potential conflict of interest.

## Publisher's note

All claims expressed in this article are solely those of the authors and do not necessarily represent those of their affiliated organizations, or those of the publisher, the editors and the reviewers. Any product that may be evaluated in this article, or claim that may be made by its manufacturer, is not guaranteed or endorsed by the publisher.
